# Salmagundi

**Published:** 1886-10

**Authors:** 


### SALMAGUNDI.

EDITED BY E. G. BETTY, D.D.S.

OCULAR DISTURBANCES CAUSED BY DENTAL IRRITATION.—At the recent Ophthalmological Congress in Paris, M. Paul Redard described a number of cases in which dental affections were evidently the source of ocular disturbance, such as glaucoma, amaurosis, amblyopia, and cloudy vision. In asthenopia, without any apparent cause, the teeth should always be examined. M. Gayet mentioned a case in which disturbance was produced by a tooth fixed on a pivot; the symptoms appeared and disappeared according as the tooth was removed or replaced. M. Fieuzal had observed so many of these cases of correlation between ocular and dental affections, that he had urged that a dental clinic should be annexed to the Quinze Vingts Hospital for blind people. M. Suarez and M. Galezowski mentioned similar facts. M. Javal mentioned a series of cases, of an inverse order, in which dental disturbance disappeared after operating for glaucoma.—*Exchange*.

### Strike the Gong.

Tremendous is the plumber's bill,
Maria, strike the gong!
The doctor makes his patient's ill,
Maria, strike the gong!
In summer girls demand ice cream,
When e're they see a snake they scream,
Their forms are seldom what they seem,
Oh!
Marıa, strike the gong!

Chicago feet are very large,
Maria, yank the gong!
The dentist first learns how to charge,
Maria, pull the gong!
A mother-in-law's an awful bore,
And though you may your wife adore
You hate the old lady more and more,
Oh!
Maria, strike the gong!

The minstrel's jokes are never new,
Maria, strike the gong!
Paragraphists are that way, too,
Maria, jerk the gong!
St. Louis girls have blanket ears,
Young widows quickly dry their tears
And marry again in about two years,
Oh!
Maria, strike the gong!

This song I sing is very nice,
Maria, strike the gong!
I'm sure you'll want to hear it twice,
Maria, strike the gong!
Maybe you won't, though—I can't tell,
If you don't want to say so—well,
Just go and buy a chestnut bell,
And
Gently jerk the gong!

—[*Somerville Journal.*

Dr. Watt has a most interesting and instructive article on "Ammonia" in the August number of the "Journal," to the doctrine of which we heartily subscribe. On page 355 occurs the following: "And right here it may not be amiss to mention that the popular notion is that the teeth are injured, or often destroyed by the medicines used during a spell of sickness; and some decline to employ regular physicians, and run after the most ignorant pretenders, believing that the quack doesn't use strong medicines, and hence will not do injury to the teeth. *It is the duty of dentists to teach their patrons, when opportunity offers, that in nearly all cases, the damage is done, if at all, by the disease.*"

We put the last clause in italics to emphasize our approval of the statement, one which we have constantly tried to beat with a stuffed club into the heads of people who are only too prone to put the blame upon the physician—the results of their own dereliction of duty. And in this connection we may say that one cannot, under such circumstances, be too careful of expressing his opinion upon the condition of any work that may be in such a person's mouth, lest we make a blunder that will do ourselves no good and may be a serious injury to the reputation of a brother practitioner.

---

Tobacco Amaurosis.—A blacksmith, aged 32, complained of failure in vision, to such an extent that he could no longer see to drive nails in shoeing; and was compelled to depend upon his sense of feeling. His health was good; he having no other complaint. Vision was found to be only one-sixth of what it should be. Things appeared to him to be covered with a dense mist. For many years he had been an excessive smoker; using the strongest tobacco to be had. Tobacco amaurosis is quite common; but usually in men beyond middle life. The prognosis is favorable, provided the habit be given up. This should be done gradually. In this case, a very few days' abstinence from tobacco caused an improvement in vision, and the man has now made material progress towards recovery. Chewing tobacco does not, in my experience, produce amaurosis.—*Dr. A. D. Williams in St. Louis Med. & Surg. Journal.*

HYDRONAPHTHOL.—We desire to call the attention of our readers to this new antiseptic, as one destined to win a far higher place than most of the new compounds recently produced by the searchers in organic chemistry.

It is non-corrosive, non-poisonous, and inodorous. Its antiseptic power is far greater than that of carbolic acid. It is not decomposed by the products of putrefaction. It is not volatile at ordinary temperatures. The vapor is not harmful when inhaled. It will not injure colors or fabrics.

It is soluble in 1,000 times its weight in water; and this strength is sufficient to indefinitely preserve organic substances from decomposition; and yet has no injurious effect on living tissues.

It will not injure the edge of the surgeon's instruments. It is freely soluble in alcohol, ether, chloroform, glycerine, benzole, and the fixed oils. Mixed with absorbent powders, like fuller's earth, it can be dusted over wounds or dressings, like iodoform: a two per cent. mixture being strong enough.

Any of the ordinary substances used in dressing wounds may be soaked in its alcoholic solution and dried: the chrystals of hydronaphthol adhering to the tissue of the dressing. It will be found of value in preserving milk, and other articles of food in hot weather, or on sea voyages. Fruit, coated with a solution in oil, may long be preserved for the use of invalids. Many other uses will suggest themselves.*—*Med. World.*

*Hydronaphthol is at present importad only by Seabury & Johnson. The price is $1.50 per lb., or 20c. per oz., post paid.

---

THE new treatment of whooping cough consists in the application with a brush, every two hours, of a one or two per cent. solution of resorcin. The applications are made to the pharynx and its supra-glottic portion, as well as to the opening of the glottis.—*N. Y. Medical Journal.*

---

A MR. CRESWELL HEWETT has made the discovery that quinine may be made by synthesis from an article which may be had in abundance in all parts of the world.

A PHYSICIAN writing on Mistakes in Practice, among other things says:—I never received any training in the extraction of teeth, and when a student in the office of my preceptor, I made a total failure at one time in trying to extract a tooth. That should have been a sufficient lesson to me, but it wasn't. I occasionally receive a call to extract a tooth. I do not own a pair of tooth forceps, and always send such calls to a dentist. One rainy night, about three years ago, I was prevailed upon to visit a lady for the purpose of extracting a tooth. I was told that the friends had a pair of forceps. The truth is, I haven't confidence enough in myself to extract a tooth. I failed, of course. I then furnished my horse and carriage, gratis, for the friends to take her to a dentist. Every failure, however small, cannot help but tarnish a man's professional reputation. I have not attempted to extract a tooth since, and when I do, "it will be a cold day." It is true that country practitioners must extract teeth, and I believe that that branch of *minor surgery* should be taught, practically, in our medical colleges. A little actual practice, under the guidance of an experienced operator, would go a good ways in establishing a professional reputation.

---

BOSTON, August 13.—On Thursday a remarkable surgical operation was performed at the Massachusetts General Hospital by Dr. Maurice H. Richardson, of this city. About a year ago John McCarthy swallowed a set of artificial teeth. The passage of food to the stomach was almost wholly prevented, the patient grew emaciated and weak, and it became evident unless relief was had he must soon die. Dr. Richardson made a transverse cut in the left side of the abdomen, through which the man's stomach was drawn out and then cut open, when by the insertion of his arm to the elbow, Dr. Richardson was able to reach and remove the teeth. The internal opening was then closed with fine silk and the stomach replaced, the external cut being also closed with stitches. The whole operation was completed in forty-five minutes. The patient is doing well, and his complete recovery is now considered little less than certain.—*Cincinnati Commercial Gazette.*

Boric Acid Tooth Powder.—For various obvious reasons, a tooth powder that is antiseptic has advantages over one that is not. Hence we give the following, which Dr. A. D. MacGregor (*Brit. Med. Jour.*, July 10,) has used for years:

Boric acid, finely powdered, 40 grains; chlorate of potassium, ʒss; powdered guaiacum, 20 grains; prepared chalk, ʒi; powdered carbonate of magnesia, to ℥j; otto of roses, half a drop. The boric acid in solution gets between the teeth and the edges of the gums, and there it discharges its antiseptic functions; the chlorate and guaiacum contribute their quota to the benefit of the gums and mucous membrane generally; the chalk is the insoluble powder to detach the particles of tartar which may be present, and the magnesia the more soluble soft powder which cannot harm the softest enamel.

---

Apone: A New Kind of Pain Expeller.—As we imagine from its counter-irritant action, it would be very likely to relieve pain, we note that the following is said to be the mode of preparation of a sort of pain-killer recommended by Poulet (*Pharm. Zeit.*) under the name of *apone:*

| | |
|---|---|
| Capsicum | 20 parts. |
| Water of ammonia | 10 parts. |
| Oil of thyme | 1 part. |
| Chloral | 1 part. |
| Alcohol, 60 per centum | 100 parts. |

Digest the capsicum with the alcohol and ammonia during four weeks, filter, and add other ingredients.

The above might be tried in acute periodontitis.

---

For the past year we have been filling the roots of pulpless teeth with iodoform and have as yet found no reason to complain. After removing the *debris* of the pulp, wash out thoroughly with alcohol and pump in with a broach a thin paste of iodoform made either with alcohol or glycerine. When the root is full, wipe away the surplus and cover with oxyphosphate. The whole operation, including the filling, may safely be done in one sitting.

EDITING a paper is a pleasant business if you like it.

If the type is large, it don't contain much reading matter.

If we publish many formulæ folks say that they are not reliable.

If we omit them, we have no enterprise or are know-nothings.

If we have a few jokes, folks say we are rattle-heads.

If we omit jokes, folks say we are fossils

If we publish original matter, they scold us for not giving selections.

If we give selections, people say we are lazy for not writing more, and giving them what they have not read in some other paper.

If we give a complimentary notice, we are censured for being partial.

If we don't, all hands say we are a great humbug.

If we remain in our office attending to our business, folks say we are too proud to mingle with other fellows.

If we go out, they say we don't attend to our business.—*Pharmaceutical Record.*

---

COCAINE SPRAY IN EPISTAXIS.—I. A., aged twenty, suffering from a severe attack of typhoid, was troubled by frequent epistaxis, and Dr. Francis L. Haynes tells us in the *Therapeutic Gazette* (July 15) that the attacks were invariably relieved by throwing into the nostrils, for a few moments, spray from a two per cent. solution of cocaine. On one occasion, copious bleeding was allowed to persist for three hours, when the spray checked it immediately. Until the patient convalesced, the spray was needed almost daily, and frequently several times a day, but the nurse never allowed more than a few drops of blood to be lost.

---

PAINFUL DENTITION.—The following is said (*L'Union Medicale*) to be an excellent preparation in painful dentition:

| | |
|---|---|
| Cocaine hydrochlorate, | |
| Sodium borate.................... | aa gr. iv. |
| Syrup of althæa.................. | ♏ 64. |
| Syrup of poppy to make....... | ♍ 100. |

A little to be rubbed on the gums several times a day.

Serious Oral Bleeding After a Leech.—In the *Vratch*, No. 12, 1886, p. 214, Dr. E. N. Mikhnevitch decribes the case of a middle-aged, moderately nourished, non-hæmophilic peasant woman, who was brought to him with all the usual symptoms of severe acute anæmia, and with incessant spitting of blood. According to her statement, having suffered for three days from toothache, she applied a leech to her gum. The leech fell off, but since then, (more than twenty-four hours), the wound was bleeding profusely all the time, in spite of the use of cold and a solution of perchloride of iron. The hemorrhage was immediately arrested by plugging with styptic cotton-wool (*gossypium ferratum.*)—*Med. and Surg. Rep.*

---

Nitrous Oxide as an Anæsthetic.—M. Laffont, in a recent communication to the Paris Societe de Biologie, stated that nitrous oxide is a most dangerous anæsthetic. He has since further prosecuted his experiments; and, at a subsequent meeting, confirmed his previous statements. He has found proof that nitrous oxide is not an anæsthetic, but an asphyxiating agent, as MM. Jolyet and Blanche have proved. When this agent is used by dentists to produce anæsthesia, hyperglycæmia and glycosuria result. M. Laffont has verified these phenomena by personal experience. He has also ascertained that in animals these results take place before anæsthesia, during the period of deep breathing.—*Exchange.*

---

The *Madrid Medical Journal* of July, 1885, reports that Miss Dolores Lleonart-y-Cassanovas, daughter of Dr. Agnew Lleonart, has just obtained, by unanimity of votes, her M. D. degree at the Barcelona University, after passing a brilliant examination. Miss Lleonart commenced her studies at the age of 8, got her B. A. degree at 13, carried off seven prizes and the highest marks in every examination, and now, at 19, her full degree in medicine and surgery. It would seem that ladies mature early in Spain.

---

The United States uses 250,000 lead pencils daily.

THE tongue is the indicator of the system. A white-coated tongue indicates febrile disturbance; a brown, moist tongue indicates disordered digestion or overloaded *primæ viæ;* a brown, dry tongue indicates depressed vitality, as in typhoid conditions and blood poisoning; a red, moist tongue indicates debility, as from exhausting discharges; a red, dry tongue indicates pyrexia, or any inflammatory fever; a "strawberry" tongue, with prominent papillæ, indicates scarlet fever or rotheln; a red, glazed tongue indicates debility, with want of assimilative power of digestion; a tremulous, flabby tongue indicates delirium tremens; hesitancy in protruding the tongue indicates concussion of the brain; protruding at one side indicates paralysis of the muscles on that side.—*Pacific Record.*

---

THE mummy of Remeses II, *the* Pharaoh who sought to slay Moses, was unwrapped on June 3rd last and the face photographed, an engraving of which appears in the *Chicago Tribune* of Sept 5th. Prof. Maspero, the Egyptologist, in describing the mummy, says: "The body is that of a vigorous and robust old man, *with white and well-preserved teeth,* white hair and eyebrows, etc." Remeses was the third King of the 19th dynasty, a man over six feet in height, of great vigor, and lived to be nearly 100 years of age.

---

ACCORDING to Dr. Ernst Freund, blood will not cogulate for a period of twenty-four hours, if it is drawn with a vessel smeared with vaseline or any oil or grease. He concludes from his experiments that, "as on the one hand the absence of adhesion of the blood to the walls of the vessels prevents coagulation, so, on the other, if any adhesion is allowed (as stirring with an unoiled glass rod) after the blood is drawn from the body, this acts as the starting point of the coagulation."

---

THE *Medical World* says that dentists in the East Indies charge from $15 to $25 for a filling of common paste (amalgam presumably). This is a chance for the overflow from our colleges.

Necrosis of the Lower Jaw Due to the Fumes of Phosphorus is unfortunately of sufficiently frequent occurrence to be scarcely worthy of note; but the following case, reported by Mr. Hutchinson, at the Clinical Society of London, possesses features worthy of mention. The patient, a lady of forty-five, after taking pills containing 1-33 grains of phosphorus t. i. d. for two years, consulted Mr. H. for necrosis. In this case, as in all others on record, carious teeth were found to exist.—*Medical World.*

---

"It is disappointing to learn that the Eucalyptus has had no visible effect on the banishment of malaria from the Roman Campagna, and that whatever has been done in that direction must be credited to drainage and the ardent rays of Old Sol." The once famous oil of Eucalyptus also seems to have gone out of fashion for dental purposes, notwithstanding the fact that it is a most excellent disinfectant.

---

Iodol has been found to be a powerful antiseptic, having an anesthetic action and promoting the granulation of wounds. It is soluble in alcohol, chloroform, ether, and slightly in olive oil. It contains nearly ninety per cent. of iodine, and is free from the disagreeable odor which characterizes iodoform.—*Weekly Medical Review.*

---

The annual production of coca leaves in Bolivia amounts to 7,500,000 lbs., of which Bolivia consumes 55 per cent., the United States and Europe, 5 per cent.; and the rest is consumed in other parts of South America. What a small proportion is devoted to legitimate scientific and medical purposes!

---

Japan publishes and supports seven medical periodicals, nine on sanitation, two on pharmacy, while seven others are devoted to various branches of science. We hope it may not be long before a dental one will appear.

---

We recently extracted three separate and distinct roots, all belonging to the first superior right bicuspid.

The British Medical Association and the International Congress.—A cablegram to the *New York Medical Journal* from Brighton, giving an account of the meeting of the British Medical Association, says: "The American delegation present, including Dr. Davis and others, made a statement before a crowded general meeting in regard to the affairs of the International Medical Congress. The delegates were received with enthusiasm, and their renewed invitation by the members of the Association to visit Washington in 1887 was cordially accepted."

---

The general object looked for in this world is to obtain the greatest possible effect with the smallest power; if so, the more simple the language, the more matter is condensed, the nearer we approach perfection.

Flourishes and flowers of rhetoric may be compared to extra wheels applied to a carriage, increasing the rattling and complexity of the machine, without adding to either the strength of its fabric or the rapidity of its course.—*Capt. Marryat.*

---

Can a Man Have a Female Complaint?—A young man entered the Dispensary of the Chicago Policlinic recently, and going up to the clerk held out one of the Dispensary circulars with the question: "Say! isn't this the hour for diseases of women?" The clerk answered in the affirmative, when the young man said: "Well! I've got a disease of a woman and want to be treated."—*Jour. Amer. Med. Assn.*

---

On Tuesday, August 31st, M. Chevreul, the French chemist and scientist, celebrated his 100th birthday. His native town, Angers, gave a procession, dinner, and fireworks in honor of the event. He is hale and hearty and works from nine to ten hours daily.

---

The tooth of a mastodon recently exhumed near Milan, Mich., measured 12¼ inches across the top, 3½ inches wide, and 7 inches deep.

---

Noah Webster's manuscript for his dictionary filled two barrels.

